# Biomarkers as Predictors of Recurrence following Curative Resection for Pancreatic Ductal Adenocarcinoma: A Review

**DOI:** 10.1155/2014/468959

**Published:** 2014-06-24

**Authors:** Sylvester N. Osayi, Mark Bloomston, Carl M. Schmidt, E. Christopher Ellison, Peter Muscarella

**Affiliations:** Department of Surgery and Center for Minimally Invasive Surgery, The Ohio State University Wexner Medical Center, Columbus, OH 43210, USA

## Abstract

Pancreatic ductal adenocarcinoma (PDA) is the fourth most common cancer causing death in the United States. Early tumor recurrence is an important contributor to the dismal prognosis. The availability of an accurate prognostic biomarker for predicting disease recurrence following curative resection will be beneficial for patient care. Most of the currently studied biomarkers remain in the investigational phase, with CA 19-9 being the only biomarker currently approved by the FDA. Herein, we review the utility of CA 19-9 and other investigational cellular, gene, and molecular tumor markers for predicting PDA recurrence following curative surgical resection.

## 1. Introduction

Pancreatic ductal adenocarcinoma (PDA) remains a lethal disease. It is the fourth most common cause of cancer-related death in the United States with an estimated incidence of 45,220 new cases and 38,460 deaths in 2013. The 5-year survival continues to be approximately 5% [1]. Surgical resection, though not without significant morbidity and mortality, provides the only chance for obtaining cure. Nonetheless, only about 20% of cases are localized and resectable at the time of presentation, and the 5-year survival following “curative” resection remains less than 25% [2].

Tumor recurrence is one of the main contributors to poor survival after curative resection of PDA. Many patients develop local recurrence and distant metastasis even following resection for early staged disease [3, 4]. Consequently, the ability to predict subgroups of patients with a high likelihood of early recurrence may be beneficial in the appropriate management of the disease. This information might be useful for selecting the timing and appropriateness of surgical intervention and neoadjuvant and adjuvant systemic chemotherapies. In addition, the considerable morbidity and mortality of major pancreatic resections could be avoided in patients who are unlikely to benefit as determined by preoperative assessment using accurate biomarkers.

The ability to successfully manage PDA is limited by the lack of accurate disease biomarkers. Tumor markers can be employed as tools for screening, diagnosis, prognosis, and surveillance. Though numerous diagnostic and prognostic PDA biomarkers have been proposed, most of them are still in the investigational phase. A comprehensive review of the literature in 2009 showed that more than 2300 papers have been published on over 2500 overexpressed genes that could all serve as biomarkers for PDA [5]. These numbers are expected to increase with ongoing findings of newly identified overexpressed genes. Despite the considerable amount of work that has been done in this area, CA 19-9 remains the only widely used biomarker in clinical practice today. Our interest here is in the prognostic utility of biomarkers as predictors of PDA recurrence following curative resection. This review aims to provide an overview of the use of CA 19-9 and other promising prognostic biomarkers (Table 1) to predict PDA recurrence following surgical resection.

## 2. Methods

### 2.1. Literature Search

**Figure 1:**
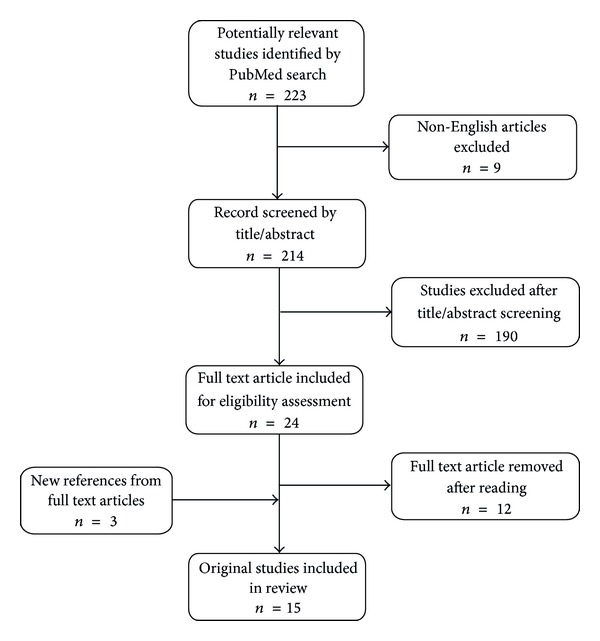
Online search strategy.

A systematic review of the PUBMED database was performed according to the Preferred Reporting Items for Systematic Review and Meta-Analyses (PRISMA) guidelines [6]. The database was searched for relevant articles published on biomarkers for predicting pancreatic cancer recurrence following curative resection. To identify relevant studies, the terms “pancreatic,” “cancer,” “adenocarcinoma,” “recurrence,” and “biomarker” were used. The search was limited to publications from 1990 to present. An initial search resulted in 223 studies. Articles in languages other than English were excluded. Next, the titles and abstracts were screened for irrelevant topics, which were excluded. Full text appraisal was performed on original studies that focused on evaluating prognostic biomarkers in patients who underwent curative resection and that addressed survival and disease recurrence as one of their endpoints. In addition, the references of the full text articles were checked for relevant publications. These studies were included in the final review.  Figure 1 shows the search strategy and the number of included original studies. A summary of the 15 original studies included in this review is shown in Table 2.

## 3. Discussion

### 3.1. Carbohydrate Antigen 19-9 (CA 19-9)

**Figure 2:**
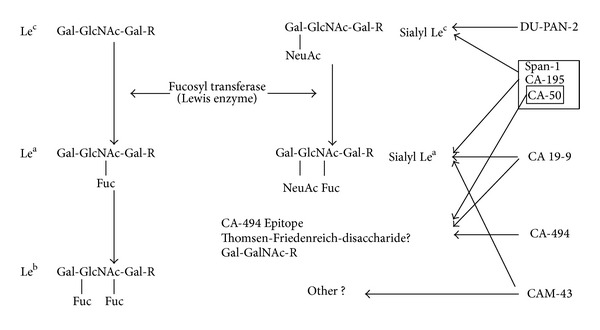
Tumor-related antigens and the carbohydrate determinants recognized by their corresponding MAbs (adapted from Muscarella II et al. [25]).

CA 19-9 is the most widely studied PDA tumor marker and the only FDA approved biomarker for PDA [22]. It was first described in 1979 using a monoclonal antibody (MAb) directed against the colon cancer cell line SW1116 [23]. It has since been most commonly utilized in the setting of pancreatic cancer [24, 25]. CA 19-9 is derived from an abnormal pathway during production of its normal equivalent, disialyl Lewis^a^. The antigenic determinant of the MAb to CA19-9 is related to a sialylated Lewis^a^ blood group and targets either Le^a+^ or Le^b+^ forms depending on the presence of specific fucosylation patterns (Figure 2). As a result, the utility of this serum biomarker is limited in patients who are Lewis blood type negative (Le^a−b−^). These individuals fail to express high level of CA19-9, even in the presence of high tumor burden. They are estimated to include 7–10% of the population [26, 27]. Another limitation of CA 19-9 is that it can be falsely elevated in the presence of biliary obstruction [26, 28]. CA 19-9 has an estimated sensitivity of 71–81% and specificity of 83–90% for the diagnosis of PDA at a cut-off level of 37 U/mL [24, 29].

In addition to the diagnostic utility of CA 19-9 in PDA, some studies have specifically assessed its ability to predict PDA recurrence following curative resection. Sugiura and colleagues evaluated preoperative CA 19-9 as a predictor of early recurrence (defined as relapse <6 months after resection) in 154 patients using a cut-off value of 100 U/mL [7]. Among 73 patients with CA 19-9 value ≥ 100 U/mL, 39 (53%) had early recurrence compared to 9 of 81 patients (11%) with CA 19-9 value < 100 U/mL (*P* < 0.001). CA 19-9 value ≥ 100 U/mL had an odds ratio of 11.2 in predicting early recurrence. Patients with CA 19-9 value < 100 U/mL had 3- and 5-year survival rates of 47.3% and 40.1% versus 21.2% and 9.4%, respectively, with a median survival time of 31 versus 16 months, compared to patients whose CA 19-9 values were ≥100 U/mL (*P* < 0.001).

In light of the limitation of CA 19-9 in the presence of biliary obstruction, some investigators have devised alternative methods of interpreting CA 19-9 levels. Kang et al. postulated that an adjusted preoperative CA 19-9 level might be a better predictor of prognosis [8]. They calculated an adjusted CA 19-9 level by dividing the serum CA 19-9 by the total bilirubin in patients with bilirubin greater than 2 mg/dL. The authors observed that patients with adjusted preoperative CA 19-9 levels > 50 U/mL had recurrence risk twice that of patients with levels < 50 U/uL (*P* = 0.027). Parameters predictive of recurrence on univariate analysis included adjusted CA 19-9 level ≥ 50 U/mL (*P* = 0.0049), peripancreatic microscopic cancer invasion (*P* = 0.0142), and lymphovascular invasion (*P* = 0.0038). Only adjusted CA 19-9 proved predictive of 12-month disease recurrence on multivariate analysis.

In addition to assessing the prognostic value of preoperative CA 19-9, some investigators have attempted to evaluate the association between postoperative CA 19-9 level and PDA recurrence. Following curative resection, it is expected that CA 19-9 levels will return to a normal range. Postoperative normalization of CA 19-9 has been linked to improved survival [9], while failure of normalization has been correlated with metastatic disease or recurrence [30]. Tian et al. assessed the prognostic attributes of elevated postoperative CA 19-9 in patients with Lewis antigen positive blood groups [9]. The group observed local recurrence and distant metastases in 6 of 11 (54.5%) patients that underwent resection. In those patients, secondary elevations in serum CA 19-9 preceded tumor recurrence by 2 to 9 months. In another study, it was observed that sustained postoperative elevation of CA19-9 level preceded clinical and radiologic detection of recurrence by 2 weeks to 5 months [31].

An investigation by Hata et al. reported a statistically significant increase in disease recurrence in patients with postoperative CA 19-9 > 37 U/mL [10]. Among this subgroup of patients, overall recurrence was 83% versus 64% (*P* = 0.002) and the rate of hepatic metastasis was 44% versus 23% (*P* < 0.0001), significantly higher in comparison to patients with postoperative CA19-9 ≤ 37 U/mL. Contrary to previously described studies [7, 8], this study did not show a statistically significant increase in overall recurrence with high preoperative CA 19-9 levels.

A novel interpretation of the postoperative CA 19-9 level that has been shown to be predictive of disease recurrence following surgery is CA 19-9 velocity. Hernandez et al. defined this as the rate at which CA 19-9 levels change over a 4-week time frame [11]. The group measured CA 19-9 velocity in 96 patients following pancreatectomy and reported it to be a better predictor of overall survival than baseline postoperative CA 19-9 (*P* < 0.001). In their study, patients with disease progression following surgery were found to have a mean velocity of 131 U/mL/4-weeks, compared to 1 U/mL/4-weeks for patients without disease progression (*P* < 0.001). The authors reported with 100% certainty that a CA 19-9 velocity of 95 U/mL/4-weeks would denote disease recurrence confirmable by radiographic imaging.

In summary, CA 19-9 is an effective marker for predicting PDA recurrence following curative resection. Preoperative CA 19-9 levels > 100 U/mL, adjusted levels > 50 U/mL, elevated postoperative CA 19-9 levels, and postoperative CA 19-9 velocity > 95 U/mL/4-weeks have all been shown to predict disease recurrence.

#### 3.1.1. Carcinoembryonic Antigen (CEA)

Prior to the availability of CA 19-9 assays, CEA was the only serum antigen used for diagnosing PDA [25]. It has since been shown to have major drawbacks in PDA diagnosis due to its much lower sensitivities and specificities in comparison to CA 19-9 [22, 32]. It has also been shown to be inferior to CA 19-9 for monitoring disease burden and surveillance following curative resection [33, 34]. However, Yasue et al. demonstrated the prognostic value of CEA in 90 patients who underwent PDA resection [35]. They reported a significant correlation between preoperative CEA level >2.5 ng/mL and poor survival. A similar correlation was reported for postoperative elevation in CEA. Despite these findings, CEA remains less informative for PDA and is currently only FDA approved as a tumor marker for colon cancer.

### 3.2. Cellular Biomarkers

#### 3.2.1. Circulating Tumor Cells (CTCs)

Tumor recurrence significantly contributes to increased mortality in patients with PDA and may be driven by early metastasis. The ability to detect metastasis at an early stage might improve prognosis following surgical resection by identifying patients who are appropriate candidates for early treatment with systemic therapy. Detection of circulating tumor cell (CTC) micrometastases in peritoneal fluid and blood has been proposed as a viable method for identifying early tumor burden. The technique employs reverse transcription polymerase chain reaction (RT-PCR). RT-PCR has been shown to be a more sensitive method for detecting metastases in the peritoneal fluid during staging laparoscopy or at the time of surgical resection as compared to conventional cytology [36–38]. CEA has been utilized as the target to identify CTCs during RT-PCR [37].

Using CEA mRNA during RT-PCR, Kelly et al. evaluated the presence of micrometastases in patients undergoing R0 resection [12]. In their study, patients with radiologic or tissue diagnosis of PDA underwent staging laparoscopy. Normal saline was introduced into the peritoneal cavity and then aspirated and sent for cytology and RNA isolation. Amplification of CEA mRNA in less than 40 cycles was deemed a positive RT-PCR. The investigators observed that, among 62 patients who underwent R0 resection, 11 (18%) had positive CEA-mRNA in their peritoneal washing by RT-PCR in the presence of negative cytology. These patients experienced higher rates of recurrence than those with negative RT-PCR (*P* = 0.003). RT-PCR positive status correlated with early recurrence (*P* = 0.01). It also correlated with decreased survival, though without statistical significance (*P* = 0.370).

In addition to peritoneal washing, CTCs have been investigated in the blood. Intraoperative measurement of CEA mRNA in venous blood by RT-PCR has shown prognostic value in detecting disease recurrence, especially in patients with high-stage disease [39, 40]. Mataki et al. investigated the significance of CTCs in the blood as an early indicator of PDA recurrence following curative resection [13]. They obtained blood sample every 3 months from 53 patients who had undergone curative resection for biliary-pancreatic cancer, 20 of whom had pancreatic cancer. The samples were evaluated for CEA mRNA expression using RT-PCR. Of the 7 PDA patients diagnosed with recurrence by radiological studies, 5 of them (71%) were blood positive for CEA mRNA. On the other hand, only 1 of the 13 PDA patients without recurrence (7.7%) had positive CEA mRNA. CEA mRNA had a sensitivity of 75% and specificity of 94% for predicting disease recurrence in this study, much better than CEA or CA 19-9 alone. CEA mRNA positive blood predated radiological diagnosis of recurrence by about 3 months. Other studies provide further clinical relevance of blood CTCs in pancreatic cancer, including CTC enrichment and detection techniques and their clinical implications [41]. Though there is a heterogeneity of publications on the utility of CTCs in predicting PDA recurrence, the above reports provide evidence of the emerging prognostic value of CTCs.

#### 3.2.2. Neutrophil-to-Lymphocyte Ratio (NLR)

Malignancies have been suggested to incite inflammatory responses in patients. It has also been suggested that recruited inflammatory cells could negatively impact survival of several types of cancer [42, 43]. Based on this knowledge, preoperative neutrophil-to-lymphocyte ratio (NLR) has been proposed as a promising predictor of survival in patients with PDA [44–46]. NLR is calculated by dividing the neutrophil count by the lymphocyte count. The sensitivity and specificity of NLR are significantly increased at levels greater than 5 [45]. Garcea et al. investigated the value of NLR in predicting prognosis following curative resection of PDA in 72 patients who underwent pancreaticoduodenectomy [14]. All patients had blood drawn preoperatively and analyzed for NLR, platelet-to-lymphocyte ratio (PLR), and C-reactive protein (CRP). At follow-up of 1 to 125.8 months, perioperative NLR among patients who experienced disease recurrence was observed to be significantly higher than that of patients without recurrence (4.7 versus 3.1, *P* = 0.02). No differences in PLR and CRP were identified between the two groups. Patients with NLR > 5 had a median disease-free survival of 12 months, significantly less than the 52 months observed in patients with NLR < 5 (*P* < 0.001). The study demonstrates the prognostic utility of this easily obtainable clinical parameter.

### 3.3. Gene Biomarkers

#### 3.3.1. P16/CDKN2A, TP53, and SMAD4/DPC4

Several gene markers have been implicated in PDA, though four of them, termed “mountain” genes, are the most commonly mutated. The genes are KRAS, CDKN2A/p16, TP53, and SMAD4/DPC4. KRAS is mutated in almost all PDA specimens. The individual and collective prognostic significance of these “mountain” genes in PDA have been the subject of numerous investigations, though fewer studies have specifically addressed their role in predicting disease recurrence. Oshima et al. evaluated the prognostic role of CDKN2A/p16, TP53, and SMAD4/DPC4 in 106 patients with PDA following surgical resection [15]. The patients had median and 5-year survivals of 22.1 months and 17.5%, respectively. Overall, 78.1% of the patients developed disease recurrence. Loss of CDKN2A/p16 was seen in 67% of cases and was associated with lymphatic invasion (*P* = 0.012), shorter overall (*P* = 0.029) and disease-free (*P* = 0.015) survivals, and distant metastases (*P* = 0.005). Abnormal TP53 was observed in 81.1% of the cases and was associated with the presence of locoregional recurrence (*P* = 0.020). SMAD4/DPC4 was absent in 54.7% of patients and this was associated with lymphatic invasion (*P* = 0.033) and shorter median overall (*P* < 0.001) and disease-free (*P* < 0.001) survivals. Noteworthy in the study was that all 6 patients with more than 5-year survival had intact SMAD4/DPC4.

Despite the above finding on SMAD4/DPC4, published reports on the utility of this gene remain contradictory. While some studies have suggested that its absence was linked with disease progression [15, 47, 48] and distant metastases [49], more recent investigations have shown no association between this tumor marker and PDA recurrence [18, 50]. Winter et al., in their evaluation of 127 patients who underwent resection for PDA, found no association between SMAD4 expression and PDA recurrence (*P* = 0.9) or death (*P* = 0.15) [50]. Though reports on the value of the “mountain genes” in predicting PDA recurrence are mixed in general, they remain viable prognostic biomarkers and deserve further investigation.

#### 3.3.2. Metastin

Metastin is a gene product of the tumor suppressor gene KiSS-1 and a ligand to the G-protein-coupled receptor GPR54 [51, 52]. PDA has been shown to express lower levels of KiSS-1 mRNA and higher levels of GPR54 mRNA in comparison to normal pancreatic tissue, suggesting the role of metastin in PDA metastasis [53]. Nagai et al. investigated the prognostic role of metastin in 53 patients who underwent curative resection for PDA. Metastin was observed to be expressed in only 24.5% of tumors [16]. These metastin-positive tumors were less likely to have recurrence (38.5% versus 70.0%, *P* = 0.04) and the presence of metastin significantly correlated with longer survival (hazard ratio = 2.1; 95% confidence interval = 1.1 − 4.7; and *P* = 0.03). Interestingly, none of the 6 patients with high plasma metastin levels in that study died during the median follow-up period of 18.5 months. Monitoring of metastin levels in resected tumor could offer prognostic value in predicting PDA recurrence, though further studies are needed to bolster this claim.

#### 3.3.3. Phosphatase and Tensin (PTEN)

Phosphatase and tensin (PTEN) is a tumor suppressor gene encoded on chromosome 10q23.3. It is one of the most commonly inactivated genes in sporadic cancers and has been implicated in several human malignancies [17]. The function of PTEN can be lost by deletion, gene silencing, mutation, or dysregulation of messenger RNA by microRNAs. Multiple studies using mouse models have shown that deletion or knockout of PTEN results in metaplasia, consequently leading to PDA [54, 55]. In about 70% of PDA, PTEN expression is either low or absent [54]. Though some studies have shown the utility of PTEN as a marker of survival in patients with hepatobiliary cancers [56], there is a paucity of studies specifically related to its usefulness for predicting PDA recurrence and survival. In one of the few related studies, Foo et al. evaluated the role of PTEN in predicting disease recurrence and survival following curative resection in 133 patients with PDA [17]. Of the 25.6% of cases with loss of PTEN, 88.2% had local recurrence or distant metastasis versus 68.7% in cases with retained PTEN expression (*P* = 0.03). In addition, 88.2% of the cases with PTEN loss had lymph node metastasis versus 71.7% of cases with retained PTEN (*P* = 0.05). Loss of PTEN correlated with decreased overall survival (32.7 ± 5.0 versus 19.9 ± 3.6 months, *P* = 0.03).

### 3.4. Molecular Biomarkers

#### 3.4.1. CX Chemokine Receptor 4 (CXCR4)

CXCR4 is a protein receptor of the CXC chemokine ligand 12 (CXCL12). It is usually overexpressed in tumor cells of epithelial origin [57]. CXCL12 is a strong chemoattractant for mature and immature hematopoietic cells. It is normally expressed in tissues like lymph node, liver and lung, and all areas susceptible to pancreatic cancer metastasis. High expression of CXCR4 in resected PDA has been associated with shorter overall survival, lymph node metastases, and liver recurrence in a small series of patients [57]. Bachet et al. investigated the prognostic value of CXCR4 in 471 patients who underwent curative resection for PDA [18]. They showed that high CXCR4 expression was significantly associated with worse outcomes in patients who did not receive an adjuvant therapy (HR = 1.69; *P* = 0.012). On multivariate analysis, high levels of CXCR4 were associated with distant recurrence (*P* < 0.001) but not with locoregional recurrence. Also, CXCR4 was shown to be a strong prognostic factor when compared with other clinical and pathological factors for predicting patients' outcome following resection. This report implicates CXCR4 as a potential biomarker of distant recurrence in PDA and as an attractive therapeutic target.

#### 3.4.2. Cathepsin B

The tendency for PDA to invade tissues and metastasize at very early stages contributes to the poor prognosis [58]. Invasion and metastasis utilize the action of proteolytic enzymes that are able to degrade components of the extracellular matrix and basement membranes [59, 60]. Cathepsin B (CTSB) is a lysosomal protease that has been shown to promote local tumor invasion and distant metastasis of PDA [58, 59, 61]. Niedergethmann et al. evaluated the prognostic significance of CTSB following R0 resection in 70 patients with PDA [19]. During the 3-year follow-up period, 58.6% of patients had disease recurrence, with early recurrence (within 6 months of resection) occurring in 18.6% of patients. CTSB immunoreactivity in the tumor cells was noted to be 95.7%. The authors observed that moderate and strong (grade 2 and 3) CTSB reactivity were associated with shorter survival (*P* = 0.005) and early postoperative recurrence (within 6 months after surgery, *P* = 0.0001) in comparison to absent or weak (grade 0 or 1) reactivity.

#### 3.4.3. Vascular Endothelia Growth Factor (VEGF)

Angiogenesis involves the development of new blood capillaries. It is an important factor in tumor invasion and metastases [62]. The process allows invasion of tumor cells through the newly formed, unorganized endothelial basement membrane [63]. Several growth factors work to promote angiogenesis, but the most potent of them is vascular endothelial growth factor (VEGF). VEGF has been shown to be prognostic in PDA, correlating with poor prognosis and local disease progression [64, 65]. It has also been shown to be associated with PDA metastasis, especially with the liver [66]. While these studies have shown the utility of VEGF as a marker of disease progression and metastasis, the question of its application as an indicator of disease recurrence remains largely unanswered. Niedergethmann et al. attempted to answer this question by investigating the correlation between VEGF expression and early recurrence following curative resection [20]. They followed 70 patients who underwent curative resection for 2 years. VEGF immunoreactivity was observed in 88.8% of the patients. There was a significant correlation between VEGF immunoreactivity and cumulative survival (*P* < 0.05), and patients with high VEGF expression had significantly higher rates of tumor recurrence within 8 months of curative surgery (*P* = 0.003). The ability to utilize VEGF in predicting early PDA recurrence will potentially identify at-risk patients for new, better-targeted adjuvant treatment. Currently, there are several preclinical and clinical trials exploring the benefits of VEGF inhibition in the treatment of PDA with some promising results [67–70]. Further discussion of these trials is beyond the scope of the current review.

#### 3.4.4. MicroRNA (miRNA)

MicroRNAs (miRNAs) are noncoding molecules involved in posttranscriptional gene regulation. Their investigation encompasses a new area of study that is showing promising clinical relevance. miRNAs are able to differentiate PDA from chronic pancreatitis through their patterns of expression [71]. The list of miRNAs implicated in PDA continues to grow and includes miR-21, 34a, 196a, 211, 217, 218, 224, and 486. miRNAs have been implicated in PDA invasion and metastasis [72, 73] and have been reported to be predictive of disease-free and overall survival [74]. Jamieson et al. investigated the genome-wide miRNA expression using microarray analysis in 48 patients who underwent curative resection [21]. After a series of confirmatory tests, the authors identified high miR-21 and low miR-34a expressions both to predict poor overall survival. Investigations on miRNAs are a novel area of focus. Further work is needed to determine their true utility for predicting PDA recurrence following resection.

## 4. Conclusion

Although surgical resection provides the only potential for cure in patients with PDA, early disease recurrence frequently limits survival. Despite numerous investigations, the goal of identifying an ideal biomarker for predicting early disease recurrence remains incompletely realized. The ability to predict patients who are at high risk for early recurrence based on tumor biomarkers can facilitate the provision of personalized treatment and may allow for improved disease survival. Presently, CA 19-9 remains the only widely used marker for PDA and represents the best prognostic biomarker for predicting disease recurrence following curative resection. Other biomarkers, such as CEA mRNA from CTCs and preoperative tumor NLR, have shown ready clinical applicability. In addition, some gene and molecular biomarkers have been investigated with promising results. Further work is needed to identify an ideal biomarker that is both accurate and feasible for predicting PDA recurrence. We remain optimistic that such a tumor marker will become available for clinical use in the near future.

## Figures and Tables

**Table 1:** Biomarkers evaluated for predicting recurrence following resection of pancreatic ductal adenocarcinoma.

Carbohydrate antigen 19-9 (CA 19-9)	
Carcinoembryonic antigen (CEA)	
Cellular biomarkers	
Circulating tumor cells (CTCs)	
Neutrophil-lymphocyte ratio (NLR)	
Gene biomarkers	
P16/CDKN2A, TP53, and SMAD4/DPC4	
Metastin	
Phosphatase and tensin (PTEN)	
Molecular biomarkers	
CX chemokine receptor 4 (CXCR4)	
Cathepsin B	
Vascular endothelial growth factor (VEGF)	
MicroRNAs (miRNAs)	

**Table 2:** Summary of original articles on biomarkers for predicting PDA recurrence following curative resection.

Author	Year	Biomarker	Cut-off level (U/mL)	Follow-up (months)	Recurrence (%)	Survival		
						Disease-free (months)	Overall median (months)	Overall 5-year (%)
Sugiura et al. [7]	2012	CA 19-9	100	NR	73.3	11	25	27.2
Kang et al. [8]	2007	Adjusted CA 19-9	50	12	69	22.6	39.6 (mean)	16.4
Tian et al. [9]	1992	CA 19-9	37	NR	54.5	NR	8.7^a^ (mean)	NR
Hata et al. [10]	2012	CA 19-9	37	NR	70	NR	16	20.3
Hernandez et al. [11]	2009	CA 19-9 Velocity	NR	41	80.2	7	12	NR
Kelly et al. [12]	2009	CTC	RT-PCR positive	10.3	NR	NR	NR	NR
Mataki et al. [13]	2004	CTCs	RT-PCR positive	49	35^b^	NR	NR	NR
Garcea et al. [14]	2011	NLR	5	NR	NR	27	35	NR
Oshima et al. [15]	2013	P16/CDKN2A TP53 SMAD4/DPC4	Presence/loss Normal/abnormal Presence/loss	NR	78.1	NR	22.1	17.5
Nagai et al. [16]	2009	Metastin	Present/absent	18.5	62.3	NR	NR	NR
Foo et al. [17]	2013	PTEN	Retained/loss	40.7	88.2^c^	12.1 ± 1.9	25.2 ± 3.0	NR
Bachet et al. [18]	2012	CXCR4	Low/high	54	65.2	14.5	30	NR
Niedergethmann et al. [19]	2004	CTSB	Grades 0 + 1 versus grades 2 + 3	36	58.6	NR	16	NR
Niedergethmann et al. [20]	2002	VEGF	Grades 0 + 1 versus grades 2 + 3	31	58.6	NR	16	NR
Jamieson et al. [21]	2012	miR-21 miR-34a	High Low	23.9 23.9	77 77	NR NR	16.5 13.4	NR NR

^a^Among cases with failed CA 19-9 normalization.

^b^Among patients with pancreatic cancer.

^c^Among cases with loss of PTEN.

U/mL, unit/milliliter; NR, not reported; CA 19-9, carbohydrate antigen 19-9; CTCs, circulating tumor cells; RT-PCR, reverse transcriptase polymerase chain reaction; NLR, neutrophil-lymphocyte ratio; PTEN, phosphatase and tensin; CXCR4, CX chemokine receptor 4; CTSB, Cathepsin B.
